# Inactivation of Heparin by Cationically Modified Chitosan

**DOI:** 10.3390/md12073953

**Published:** 2014-06-30

**Authors:** Barbara Lorkowska-Zawicka, Kamil Kamiński, Justyna Ciejka, Krzysztof Szczubiałka, Magdalena Białas, Krzysztof Okoń, Dariusz Adamek, Maria Nowakowska, Jacek Jawień, Rafał Olszanecki, Ryszard Korbut

**Affiliations:** 1 Chair of Pharmacology, Jagiellonian University Medical College, 16 Grzegórzecka Str., Cracow 31-531, Poland; E-Mails: mmjawien@cyf-kr.edu.pl (J.J.); mfolszan@cyf-kr.edu.pl (R.O.); mfkorbut@cyf-kr.edu.pl (R.K.); 2 Faculty of Chemistry, Jagiellonian University, 3 Ingardena Str., Cracow 30-060, Poland; E-Mails: kaminski@chemia.uj.edu.pl (K.K.); justyna.ciejka@gmail.com (J.C.); szczubia@chemia.uj.edu.pl (K.S.); nowakows@chemia.uj.edu.pl (M.N.); 3 Department of Pathomorphology, Jagiellonian University Medical College, 16 Grzegórzecka Str., Cracow 31-531, Poland; E-Mails: mbialas7@gmail.com (M.B.); mpokon@cyf-kr.edu.pl (K.O.); mnadamek@cyf-kr.edu.pl (D.A.)

**Keywords:** chitosan, HTCC, unfractionated heparin, heparin antidote, protamine, coagulation, hepatotoxicity

## Abstract

This study was performed to evaluate the ability of *N*-(2-hydroxypropyl)-3-trimethylammonium chitosan chloride (HTCC), the cationically modified chitosan, to form biologically inactive complexes with unfractionated heparin and thereby blocking its anticoagulant activity. Experiments were carried out in rats *in vivo* and *in vitro* using the activated partial thromboplastin time (APTT) and prothrombin time (PT) tests for evaluation of heparin anticoagulant activity. For the first time we have found that HTCC effectively neutralizes anticoagulant action of heparin in rat blood *in vitro* as well as in rats *in vivo*. The effect of HTCC on suppression of heparin activity is dose-dependent and its efficacy can be comparable to that of protamine-the only agent used in clinic for heparin neutralization. HTCC administered *i*.*v*. alone had no direct effect on any of the coagulation tests used. The potential adverse effects of HTCC were further explored using rat experimental model of acute toxicity. When administered *i*.*p*. at high doses (250 and 500 mg/kg body weight), HTCC induced some significant dose-dependent structural abnormalities in the liver. However, when HTCC was administered at low doses, comparable to those used for neutralization of anticoagulant effect of heparin, no histopathological abnormalities in liver were observed.

## 1. Introduction

Heparin, usually used in cardiovascular surgery to prevent thromboembolism, often leads to a high incidence of bleeding complications. To reverse its anticoagulant action, protamine sulfate is routinely used. Protamine consists of a group of heterogeneous cationic peptides obtained from fish and its antiheparin activity results from the attractive electrostatic interaction with anionic heparin [1]. It is a relatively safe drug; however, its administration is occasionally associated with severe, life-threatening reactions, including significant systemic hypotension, severe pulmonary hypertension, anaphylactic shock, fatal cardiac arrest as well as thrombocytopenia [2]. Although adverse events after protamine administration might be associated with significant morbidity and mortality, there is a lack of clinically reliable alternative substance for heparin neutralization.

In an attempt to address this clinical problem, we tested a modified chitosan—a polymer derived from chitin through partial deacetylation—as a potentially effective heparin antagonist. Chitosan, as a cationic polysaccharide, can interact with negatively charged (macro)molecules. This feature was essential for our concept that chitosan can interact with heparin. Chitosan is one of the most promising polymers in the biomedical field because of its biocompatibility and biodegradability [3,4]. In recent years, chemically modified chitosans, developed in order to improve the properties of the native chitosan for oral drug delivery, have gained increasing attention [5,6,7]. Moreover, other beneficial properties such as antitumor, hypocholesterolemic, weight lowering, antioxidant, neuroprotective, and antimicrobial presented it to nutritional health supplements [7,8,9].

However, the main limitations on use of chitosan in several applications are its high viscosity and low solubility at neutral pH. Thus, *N*-(2-hydroxypropyl)-3-trimethylammonium chitosan chloride (HTCC), a bioactive cationically modified chitosan has been obtained by the chemical substitution of chitosan with glycidyltrimethylammonium chloride (GTMAC) containing cationic ammonium groups [10]. The main rationale for carrying out chemical modification of the chitosan molecule was to obtain a compound with good solubility at physiological pH—ideally at pH = 7.4 which is the value typical for blood, allowing to perform animal study with the use of *i*.*p*. and/or *i*.*v*. routes of administration. Cationically modified (quaternized) chitosan is a beige-colored, solid-stated, stable at room temperature substance that can be powdered. HTCC shows good solubility at physiological pH and displays a high efficiency of heparin binding. Finally, it was proved that in model buffers at neutral pH HTCC forms complexes with heparin molecules [10,11]. Therefore, the aim of our study was to test the ability of HTCC to neutralize anticoagulant activity of heparin in rat blood *in vitro* and *in vivo*. 

## 2. Materials and Methods

### 2.1. Materials

Heparin sodium injection (5000 USP units/mL) was purchased from Polfa (Warsaw, Poland). Protamine sulfate salt was purchased from Sigma-Aldrich (St. Louis, MO, USA). HTCC was prepared from native chitosan (Sigma-Aldrich, St. Louis, MO, USA) according to the procedure described previously [10]. The solutions of heparin, protamine and HTCC were prepared in 0.9% NaCl.

### 2.2. Animals

The experiments were carried out using Wistar male rats. The animals were kept in plastic cages in a room temperature with a 12:12 light:dark cycle. They were fed with a standard chow diet and had free access to water. All experiments were conducted according to the Guidelines for Animal Experiments, Jagiellonian University, Poland. The study was approved by the First Local Ethical Committee on Animal Testing at the Jagiellonian University in Krakow, Poland (decision 74/2010 valid from 7 July 2010 to July 2013; decision Zi/497/2009 valid from 9 February 2009 to 9 February 2014).

### 2.3. In Vitro Experiments

The experiments were carried out on blood of rats weighing 430–495 g. Animals were anesthetized with 30 mg/kg sodium pentobarbital and carotid artery was cannulated. Arterial blood was collected into plastic tubes containing 3.18% sodium citrate (9:1, v:v blood to anticoagulant). Each sample, containing 1.5 mL of blood, was treated for 5 min with heparin alone at concentrations from 0.5 to 3.0 IU per 1.0 mL of blood. Blood was also treated with heparin in combination with HTCC or protamine at the concentration adjusted to neutralize heparin activity, *i.e.*, 1.4 mg of HTCC and 1.2 mg of protamine for each 120 IU (about 1 mg) of heparin, the proportions determined previously using model buffers [10]. Then, the samples were mixed gently. In control samples, 0.9% NaCl was added to blood. Blood was also treated with HTCC or protamine alone at concentrations corresponding to those used in experiments with heparin. For measurements of blood clotting parameters, blood plasma was separated by centrifugation at 1200× g for 5 min at 4 °C, frozen and stored at −80 °C until coagulation assays were performed. Each experiment was performed three times.

### 2.4. In Vivo Experiments

*In vivo* experiments were performed on animals weighing 310–375 g. Animals were anesthetized with 30 mg/kg sodium pentobarbital and femoral vein and carotid artery were cannulated. The animals were divided into six experimental groups. 

Group 1: control rats (*n =* 3) that received physiological saline *i*.*v*. (500 μL of 0.9% NaCl). 

Group 2: rats (*n =* 3) pretreated with heparin *i*.*v*. (dissolved in saline, 1 IU/g body weight). 

Group 3: rats (*n =* 6) pretreated *i*.*v*. with heparin (1 IU/g body weight) and next with HTCC *i*.*v*. at a dose of 1.4 mg per each 120 IU of given heparin.

Group 4: rats (*n =* 6) were given *i*.*v*. injection of HTCC at a dose adjusted to neutralize heparin. 

Group 5: rats (*n =* 3) pretreated *i*.*v*. with heparin (1 IU/g body weight) and then injected with protamine *i*.*v*. at a dose of 1.2 mg per each 120 IU of pre-injected heparin. 

Group 6: rats (*n =* 3) injected with protamine *i*.*v*. at a dose adjusted to neutralize heparin activity.

The dose of heparin administered to rats was 1 IU/g body weight. The dose of protamine was based on the *in vitro* established neutralization dose, *i.e.*, 1.2 mg:120 IU (protamine:heparin). The dose of HTCC administered to achieve a complete heparin neutralization was determined earlier in model buffers (PBS, 0.9% NaCl) and was based on the proportion 1.4 mg:120 IU (HTCC:heparin), thus the total HTCC dose administered *i*.*v.* was 11.6 mg/kg. These compounds were administered intravenously into femoral vein and blood samples were collected from carotid artery into plastic tubes containing 3.18% sodium citrate after 30 and 60 min. Before each tested substance administration control samples were collected. Blood samples were also collected 5 min after heparin administration. For the measurements of hemostatic parameters blood plasma was obtained in the same manner as in the *in vitro* tests.

### 2.5. Hemostatic Parameters

The coagulation screening tests, *i.e.*, activated partial thromboplastin time (APTT), prothrombin time (PT), and fibrinogen (Fb) concentration were performed with commercially available coagulometer (Hemostasis Analyzer, Start-4^®^, Diagnostica Stago, Roche Diagnostics) by modifications of the conventional clinical procedures [12,13]. APTT and PT values are represented in seconds and Fb concentration is expressed as mg/dL.

### 2.6. Pathomorphological Examination of the Liver

The experiments were carried out on rats weighing 200–265 g. Rats were randomly divided into four groups (control group and three groups injected *i*.*p*. with various doses of HTCC). Each group consisted of 3 animals. The doses were 25, 200 and 500 mg/kg body weight *i*.*p*. for HTCC dissolved in physiological saline; 500 μL of 0.9% saline was injected as a control. The survival time of each animal was recorded for up to 24 h. Then animals were anesthetized with 30 mg/kg sodium pentobarbital and sacrificed by cervical dislocation. Livers of rats were fixed by the pulsating-perfusion method. At first, blood was washed out with saline, then 4% buffered formalin was injected. A portion of liver tissue was taken for histological evaluation. Liver tissue samples were embedded in paraffin blocks after routine tissue preparation procedures (dehydration in alcohol, clearing in xylitol), cut into 5 μm thick sections with a microtome blade. Sections from the liver of each animal involved in the study were stained with hematoxylin and eosin for general histological investigations (for evaluation of necro-inflammatory grading), and with oil red O for the evaluation of fatty droplets. 

### 2.7. Statistical Analysis

Data were expressed as the mean ± SEM. Statistical significance and differences among groups were determined by ANOVA one-way test. *p <* 0.05 values were considered statistically significant. 

## 3. Results

### 3.1. Effect of HTCC on Hemostatic Parameters of Rat Blood in Vitro

**Figure 1:**
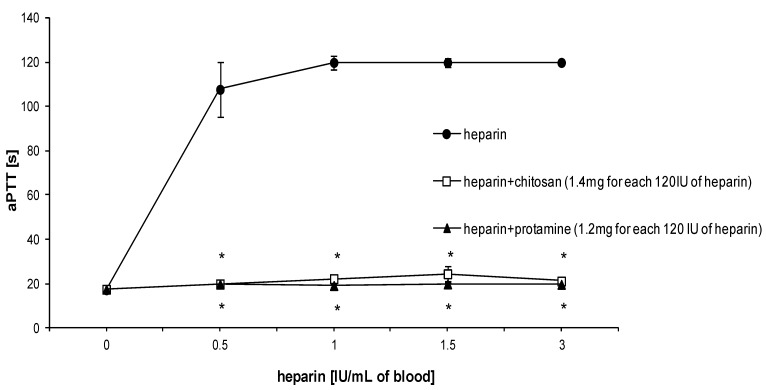
HTCC and protamine reverse the anticoagulant effect of heparin as measured by APTT in rat blood *in vitro* (*n =* 3 experiments for each point), results are expresses as mean ± SEM, * *p <* 0.05 *vs*. heparin.

**Figure 2:**
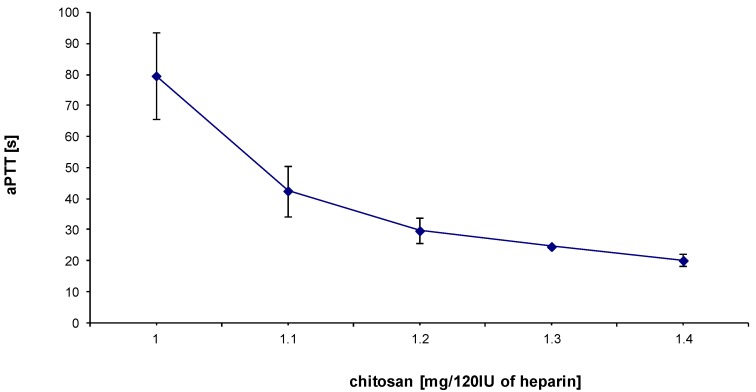
Effect of incremental concentration of HTCC on reversal of heparin activity (heparin used at a dose of 3 IU per 1 mL of blood sample) in rat blood *in vitro*. APTT values are expressed as mean ± SEM and 6 samples were used for each plotted data point.

**Figure 3:**
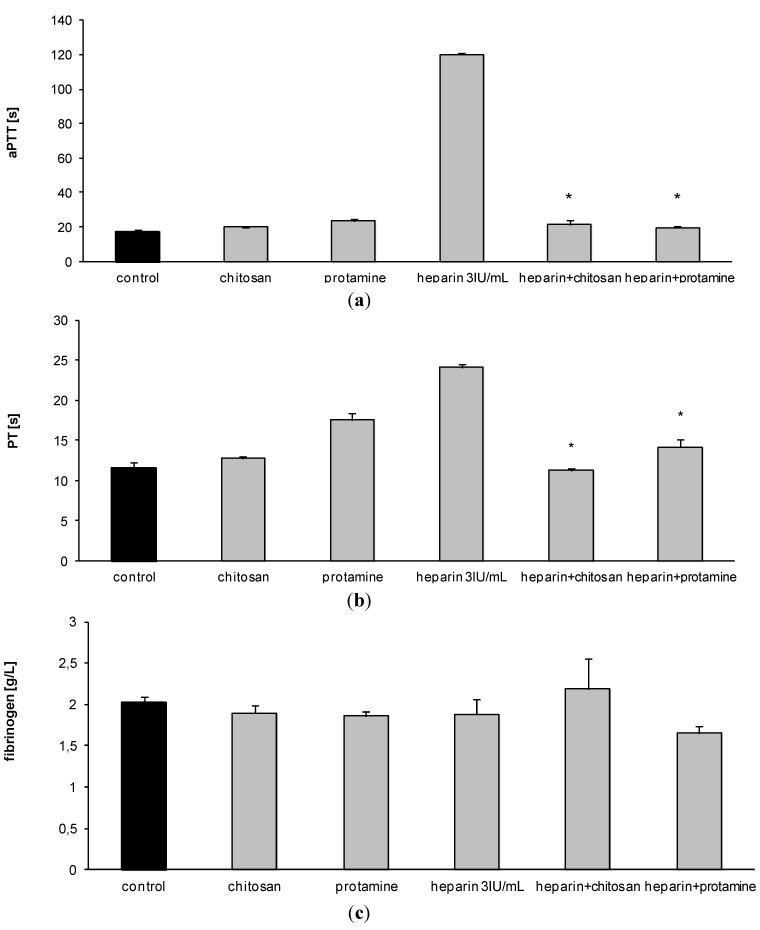
The influence of heparin, HTCC, and protamine on APTT (**a**); PT (**b**) and fibrinogen (**c**) levels in rat blood *in vitro* (results are expressed as mean ± SEM from *n =* 6 experiments; * *p <* 0.05 *vs*. heparin).

Mean APTT value of rat blood in control experiments was 17.2 s ± 1.03 s (*n* = 3). The administration of heparin resulted in an increase of APTT level, observed for every half unit of heparin added (Figure 1). On the basis of these results, we chose a dose of 3 IU of heparin/mL of blood to study the effect of HTCC on hemostatic parameters. The selected dose of heparin was found to be suitable for stable and reproducible APTT elevation. HTCC caused a dose-dependent decrease in APTT level of heparinized rat blood (Figure 2). The most effective dose of chitosan was 1.4 mg per 120 IU of heparin, as determined earlier for model buffers [10]. The effect of HTCC administration on APTT level reduction was comparable to that produced with protamine (Figure 3a). Neither modified chitosan nor protamine given alone influenced the APTT level, as compared to control (Figure 3a). HTCC decreased PT in heparinized blood and this effect was similar to that produced by protamine (Figure 3b). In contrast to protamine, HTCC alone did not influence PT level (Figure 3b). As shown on Figure 3c, the addition of HTCC to heparinized blood resulted in a slight increase of fibrinogen concentration, however, this effect was not statistically significant.

### 3.2. Hemostatic Effects of HTCC in Rats in Vivo

**Figure 4:**
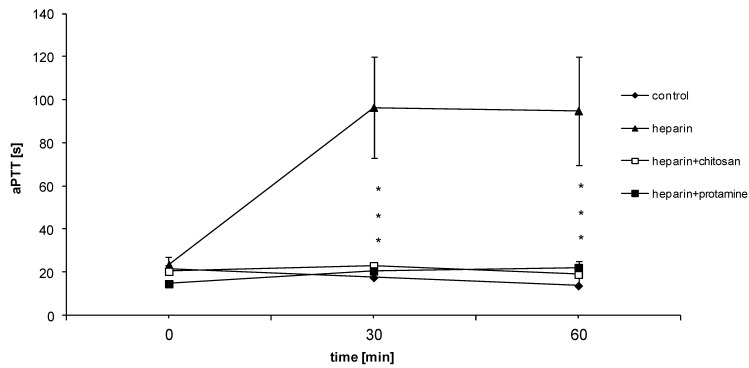
Comparison of the effects of HTCC and protamine on APTT in blood *in vivo* (the plotted data are expressed as mean ± SEM from *n =* 3–6 experiments, * *p <* 0.05 *vs*. heparin).

**Figure 5:**
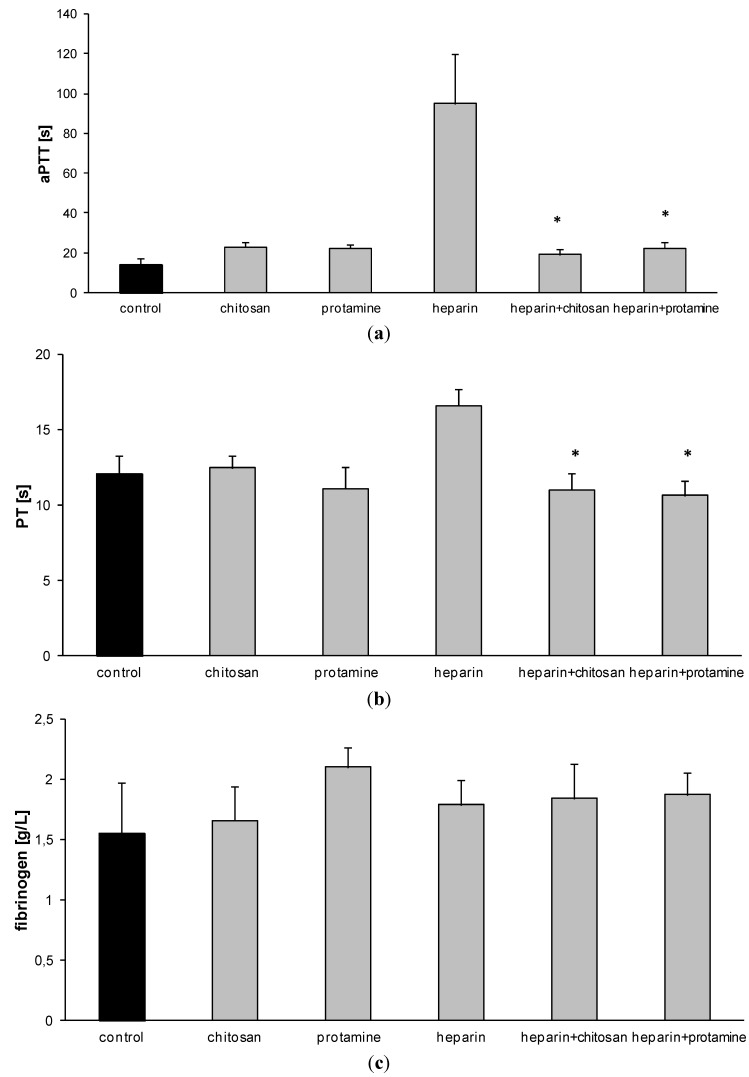
Effect of HTCC and protamine on coagulation tests: APTT (**a**); PT (**b**); fibrinogen concentration (**c**) in blood *in vivo* at 60 min after HTCC administration. The data are expressed as mean ± SEM (*n =* 3–6), * *p <* 0.05 *vs*. heparin).

There was no significant difference in APTT level in the control group during the evaluation period (0, 30 and 60 min) (Figure 4). After *i*.*v*. administration of heparin APTT was significantly increased, reaching maximal level (84.7 s ± 13.53 s) after 30 min (Figure 4). In rats pretreated with heparin, after HTCC administration the most significant drop of APTT and PT level was achieved just after 30 min and was still observed at 60 min after injection (Figure 4). Compared to the control group, HTCC as well as protamine injected alone, did not influence APTT or PT level (Figure 5a,b). As shown on Figure 5c, only protamine induced slight increase in fibrinogen level compared to the control group, but this effect was not statistically significant.

**Figure 6:**
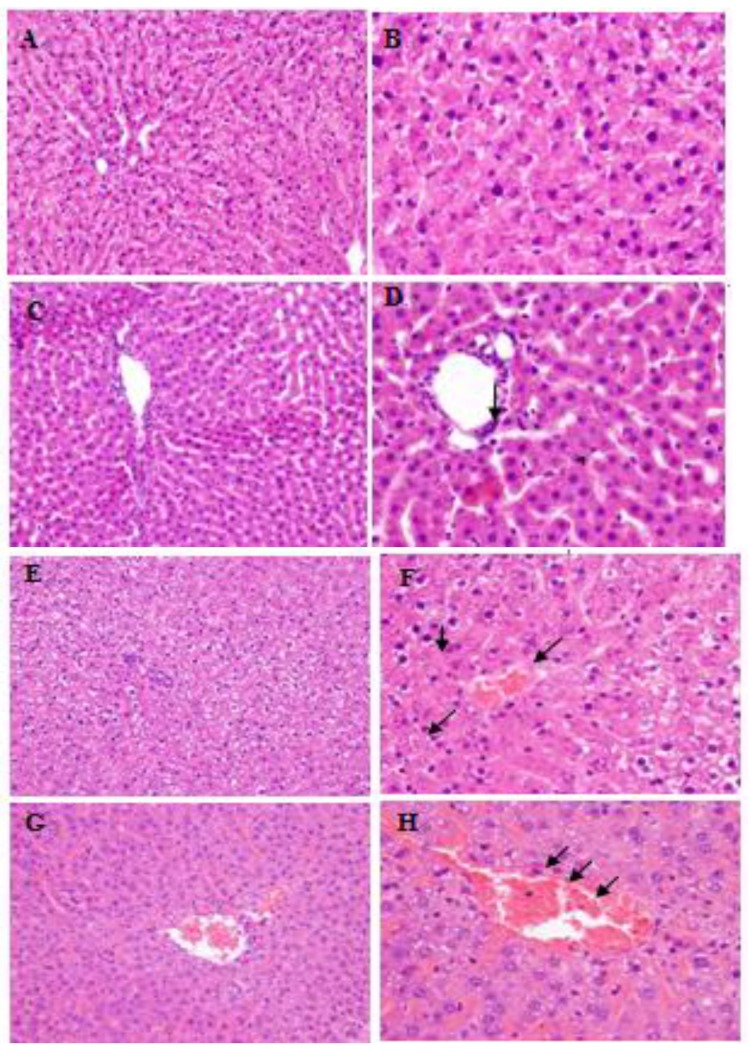
Changes of histopathological image of rat liver after *i*.*p*. administration of HTCC (hematoxylin and eosin staining). (**A**,**B**) Photomicrographs of control rat liver. The lobular structure of the liver is preserved. Portal tracts of normal size, without increase of collagen fibers and without inflammatory infiltrates. Proper bile ducts are present in all portal tracts. The structure of the cytoplasm is preserved in all hepatocytes. No signs of fatty change (original magnification A × 20, B × 40); (**C**,**D**) Photomicrographs of rat liver after *i*.*p*. administration of 25 mg/kg of HTCC. The lobular structure of the liver is preserved. Portal tracts of normal size, without increase of collagen fibers and without inflammatory infiltrates. Proper bile ducts are present in all portal tracts. Microvesicular fatty change concentrated around central veins (zone 3 of Rappaport) is seen in about 40% of hepatocytes (original magnification C × 20, D × 40); (**E**,**F**) Photomicrographs of rat liver after *i*.*p*. administration of 200 mg/kg of HTCC. The lobular structure of the liver is preserved. The signs of congestion are visible. Portal tracts of normal size, without increase of collagen fibers and without inflammatory infiltrates. Proper bile ducts are present in all portal tracts. The structure of the cytoplasm is damaged in majority of hepatocytes. Isolated necrosis of hepatocytes mainly in the perivenular zone and sparse binucleated hepatocytes are present. No signs of fatty change (original magnification E × 20, F × 40); (**G**,**H**) Photomicrographs of rat liver after *i*.*p*. administration of 500 mg/kg of HTCC. The lobular structure of the liver is preserved. The signs of severe congestion are visible. Portal tracts of normal size, without increase of collagen fibers and without inflammatory infiltrates. Proper bile ducts are present in all portal tracts. Microvesicular fatty change concentrated around central veins (zone 3 of Rappaport) is seen in about 80%–90% of hepatocytes (original magnification G × 20, H × 40).

### 3.3. Histopathological Findings

All rats survived *i*.*p*. administration of HTCC. The histopathological assessment of liver was performed for all experimental groups. Rats in the saline control group showed normal, well defined liver histological structures without any signs of vascular or inflammatory changes (Figure 6A,B). All tested doses of HTCC did not influence the lobular structure of the liver and also portal tracts were intact without increase of collagen fibers and without inflammatory infiltrates. In histopathological evaluation of the liver tissue samples, there were no significant changes between control group and group administered with 25 mg/kg dose of HTCC. However, some fatty changes were observed after HTCC administration (Figure 6C,D). The 200 and 500 mg/kg doses of HTCC induced liver damage (Figure 6E–H). We observed some dose-dependent structural changes, like microvesicular fatty changes concentrated around central veins (zone 3 of Rappaport) in 40% of hepatocytes in group treated with 25 mg/kg of HTCC to 80%–90% of hepatocytes in the group treated with 500 mg/kg. However, in our study total dose of HTCC used *i*.*v*. in rats for reversing heparin activity was 11.6 mg/kg and this dose appeared to be non-toxic.

## 4. Discussion

In this study, we have investigated antiheparin potential of HTCC by measuring its effects on blood hemostatic parameters in rats. It was shown earlier that HTCC had the ability of forming a complex with heparin [10]. However, this was only demonstrated in model buffers. Here, for the first time we have found that HTCC may neutralize anticoagulant activity of heparin also in blood, as revealed by significant APTT reduction in rats *in vitro*. Moreover, we also tested *in vivo* anticoagulant activity of HTCC. We have provided clear evidence that in rats pretreated with heparin, intravenous administration of HTCC significantly lowers APTT level in a dose-dependent manner. In our experiments in rats the administration of HTCC at a dose of 1.4 mg per 120 IU of heparin (about 1 mg of heparin) effectively leads to the normalization of APTT. The pharmacological antiheparin activity of the studied polymer is comparable to that of protamine—the only agent used so far in clinic for heparin neutralization. 

To our knowledge, there are no reports, describing the effect of chitosan on coagulation parameters of heparinized blood *in vivo* and a number of significant restrictions to study this compound *in vivo* result from its unfavorable physicochemical properties. It is well known, that high molecular weight and high viscosity of chitosan may limit its use *in vivo* [14]. Non-modified chitosan is soluble at acidic pH that makes it difficult to use it intravenously [15]. Therefore, several chemical methods have been developed to prepare safe and biologically active low molecular weight compounds. However, they are expensive since their synthesis is complicated and laborious [16,17,18]. That is why there is only a little data, regarding the effect of chitosan and its derivatives having different physicochemical properties on the blood coagulation *in vivo*. HTCC tested in this study has a good solubility at neutral pH (previously shown in model buffers) and thus it is possible to perform *in vivo* experiments involving its parenteral administration [10].

It is important to mention that chitosans with their diverse chemical structure show a variety of biological activities, and not all of these biological activities may be at the same time shown in all types of chitosans [4,19]. Chitosan has three types of reactive functional groups: an amino/acetamido group as well as primary and secondary hydroxyl groups. Many novel chitosan derivatives have been recently obtained by chemical modification of these groups [7]. However, the distribution of the acetyl groups along the chitosan molecules seems to be of crucial importance for its interaction with the biological systems [17]. In some cases the molecular weight plays a dominant role [19,20]. Regarding the effects of different chitosans on hemostasis, there are some reports of an *in vitro* and *in vivo* experiments and they are focused on their influence on platelet function, red blood cells aggregability, or blood coagulation time (BCT) [21,22,23]. It was shown that chitin and chitosan suspensions at concentrations up to 1 mg/mL reduced BCT significantly in a concentration-dependent manner and enhanced the release of some platelet factors (e.g., PDGF-AB, TGF-β1) [23]. On the other hand, it was found that chitosan is an effective inductor of platelet adhesion and aggregation and the mechanism of its action includes intracellular free Ca^2+^ mobilization and enhancing expression of platelet GPIIb/IIIa complex, increasing the expression of αIIbβ3 integrins and P-selectin [7,21,24,25]. These results indicate that hemostatic activity of chitosan may be independent of the classical coagulation cascade. However, it is also believed that coagulation of blood may be activated by chitosan and it appears to go via activation of platelets with the direct participation of plasma and extracellular matrix proteins [24]. It was already revealed that upon exposure of whole blood to chitosan the thrombus is formed very rapidly [7,26]. 

Zhang *et al*. measured in blood *in vitro* coagulative activity of chitosan hydrochloride with different degree of acetylation and molecular weight, using tube and capillary tube methods [7]. They found that the solution of chitosan hydrochloride with higher degree of acetylation (DA) and molecular weight (Mw) exhibited better hemostatic activity while for the powdered form of chitosan the effect of DA and Mw was only slight. Moreover, the comparative study carried out using solid chitosan and chitosan acetic acid physiological saline solution revealed disparate hemostatic mechanisms produced by different chitosans. The addition of chitosan acetic acid physiological saline solution to blood resulted in the aggregation and deformation of erythrocyte membranes. However, solid-state chitosan with a high deacetylation degree bound more platelets and was more hemostatic [7]. The end-point of these cited studies was the analysis of platelet and/or erythrocyte aggregatory function, however, there is still no assured verdict of the mechanism by which these different chitosans act to stop bleeding. Some researchers were convinced that chitosan could induce clot formation in the absence of platelets, thus postulating the platelet-independent mechanism [27]. Earlier it has been found that coagulation factors could not be activated by chitosan and its derivatives [27,28]. It should also be recalled that the lack of direct relation of chitosan with the intrinsic or extrinsic pathways of coagulation was observed as often [7,27] as that chitosan film may activate the coagulation complementary systems [24]. 

Our *in vivo* experiments in rats revealed that HTCC administered separately at doses adjusted for complete binding of heparin did not influence coagulation factors as evidenced by unchanged APTT, PT, and fibrinogen level. Thus, here we suggest that HTCC is a neutral and biocompatible polymer and it does not affect classical coagulation cascade.

An organ damaged during *in vivo* experiments in rabbits revealed that chitosan could promote rapid blood clotting in lung, spleen and kidney injuries, and also reduces the amount of bleeding, exerting a substantial *in vivo* hemostatic effect [7]. However, all of these experiments were performed in the absence of exogenous heparin. Even in a therapeutically anticoagulated (heparinized) rabbit model, chitosan treatment could effectively bring bleeding time within the normal range [29]. Thus, the properties of chitosan to clot blood rapidly has gained its approval for use in internal bandages and other hemostatic agents. So far, only in its chemically purest form chitosan has an internal hemostatic dressing potential [7].

As revealed earlier in model buffers, HTCC is able to bind not only unfractionated heparin, but also low molecular weight heparins (LMWHs) [10]. This unexpected observation will be elucidated *in vivo*, since until now, there is not known antidote for LMWHs. It seems that cationic modification of chitosan may be responsible not only for a good solubility at neutral pH, but also for the increase of its chemical binding force both to heparin as well as to a variety of its derivatives [10]. 

At the same time, however, it cannot be excluded that chitosan modification leads also to its increased binding to other blood components (e.g., blood proteins, erythrocytes, platelets). Unlike chains of most of polysaccharides, the chitosan macromolecules are positively charged and this property may be responsible for many biological activities related to binding to negatively charged surfaces. Indeed, it was already found that large amounts of fibrinogen and other plasma proteins may be bound to chitosan [24]. According to our experiments, the level of fibrinogen, as measured both *in vivo* and *in vitro* as well as with or without the pretreatment with heparin, was not affected by the administration of HTCC. Till now we have not studied the influence of HTCC on other blood components. 

The term “chitosan” represents a large group of structurally different chemical entities obtained during the deacetylation of chitin under treatment with strong aqueous alkali. It is known that deacetylation degree is one of the most important features, which could influence biological activity of chitosan. Generally, the level of chitosan deacetylation is 70%–90% and it is thought that 70% deacetylation degree (DD 70%) is better and safer [5,15,30]. However, the higher acetylation, the lower solubility and higher possibility to induce an acute inflammatory response. 

On the other hand, it was reported that chitosans with acetylation degree higher than 35% show low toxicity [31]. In our study the acetylation of HTCC was found to be 21.1% from the elemental analysis (DD 78.9%). Since it is known that the cationic modification increases also zeta potential (a measure of the magnitude of the electrical charge at the double layer) that could lead to increasing cellular toxicity, we decided to test safety of *in vivo* administration of HTCC using a rat model of acute toxicity. We found that chitosan samples are subjected to particularly rapid plasma clearance after *i*.*v*. injection with good distribution to all organs and high concentration in the liver [32].

Based on histopathological analysis, we have found the features of steatosis in response to *i*.*p*. administration of tested substance in a dose of 25 mg/kg. After *i*.*p*. administration of HTCC in 200 and 500 mg/kg/day doses, we found some features of the liver damage (microvesicular fatty changes concentrated around central veins, the signs of severe congestion). These findings are consistent with those from other research which found hepato-nephrotoxicity induced by chitosan administration in mice [33]. Moreover, some studies regarding the interaction between human liver cells and chitosan nanoparticles in cell culture tests showed necrotic and autophagic cell death, possibly caused by the cell membrane damage by chitosan given at concentrations above 0.5% *w/v* [34]. It was also observed that oral 150 and 300 mg/kg doses of chitosan caused significant increase in the levels of liver enzymes in mice [33]. However, it was shown that chitosan may prevent liver injury. Some studies demonstrated that chitosan decreased plasma level of ALT and AST in the rat model of steatohepatitis or obesity and possesses the hepatoprotective activity [35,36,37]. It is well known that quaternized chitosans are non-cytotoxic and have many beneficial effects [10]. On the other hand, not all chitosan derivatives proved to be nontoxic—e.g., tertiary amines are less cytotoxic than primary ones as found by in study with Monomac cells [38].

The *i*.*v*. dose of HTCC used in rats to reverse heparin activity was 11.6 mg/kg. HTCC at the above dose is safe and, as revealed by the observation of the immediate normalization of APTT, it is a very strong heparin complexing agent. This phenomenon appears to be analogous to heparin complexation by protamine. Although chitosan has profound application in many fields of life, only several articles concern its harmful side effects. Unquestionably, chitosan could induce different dose-dependent organ damages [33,39]. Thus, special care should be taken especially when long term use of chitosan would be taken into account.

During the last decade, several alternative methods were investigated for the reversal of heparin-induced anticoagulation. As an alternative to protamine administration a heparin removal device that uses extracorporeal circuit with plasma separation and poly-l-lysine affinity adsorption was proposed [40]. Methylene blue and vancomycin were also studied as heparin antidote. However, methylene blue produced incomplete reversal, and in comparison to protamine and vancomycin, it is toxic and ineffective. It seemed that for patients with protamine allergy hexadimethrine could be an alternative for heparin neutralization. However, some animal studies revealed its renal toxicity [41]. Some studies indicate that heparinase is highly effective in eliminating the anticoagulant activity of heparin *in vitro* in plasma from cardiac surgical patients [42]. On the other hand, heparinase used for heparin anticoagulation reversal in patient after aorto-coronary bypass graft surgery has inferior safety profile, compared to protamine [43]. In addition, platelet factor 4 is a very promising compound as a heparin-binding protein and was used safely in recombinant form in patients after cardiac catheterization [44]. Likewise, concatemeric heparin-binding peptides are potential candidates for the clinical reversal of heparin in humans [45]. In the above context it should finally be noted that HTCC may represent a completely novel class of heparin-neutralizing agents.

There are also some limitations to the present study that we want to underline strongly at the end of this discussion. We used APTT and PT as the end-point for heparin reversal and these are routinely measured standard parameters of blood coagulation [46,47]. However, there are data supporting also the application of anti-Xa, anti-IIa, and thrombin generation assays for anticoagulation measuring [13,48,49]. However, as yet there is no gold standard for the determination of complete heparin activity reversal, especially in animal studies [12,50]. The methods and reagents used in our study are particularly suitable for basal determination of functioning of extrinsic and intrinsic pathways of coagulation system. It seems important to test the participation of other elements of coagulation system, e.g., a function of blood platelets, red blood cells and vascular wall (endothelial function), in response to HTCC. Thus, further studies will certainly be needed.

## 5. Conclusions

In summary, HTCC is very effective in reversing heparin-induced anticoagulation both *in vitro* and *in vivo* in rats and its potency is comparable to that of protamine. As revealed by the anticoagulation measurements and histopathological results HTCC, when administered alone at doses adjusted for effective heparin complexation, is non-toxic and safe. This study for the first time shows the importance of cationically modified chitosans for potential development of a qualitatively new class of anti-heparins. 
